# Role of Cancer-Associated Fibroblast in Gastric Cancer Progression and Resistance to Treatments

**DOI:** 10.1155/2019/6270784

**Published:** 2019-06-09

**Authors:** In-Hye Ham, Dagyeong Lee, Hoon Hur

**Affiliations:** ^1^Department of Surgery, Ajou University School of Medicine, Suwon, Republic of Korea; ^2^Brain Korea 21 Plus Research Center for Biomedical Sciences, Ajou University, Suwon, Republic of Korea; ^3^Department of Biomedical Science, Graduated School of Ajou University, Suwon, Republic of Korea

## Abstract

Although the survival of gastric cancer (GC) patients has gradually improved, the outcomes of advanced GC patients remain unsatisfactory despite standard treatment with conventional chemotherapy or targeted agents. Several studies have shown that cancer-associated fibroblasts (CAFs), a major component of tumor stroma in GC, may have significant roles in GC progression and resistance to treatments. CAFs are a major source of various secreted molecules in the tumor microenvironment, which stimulate cancer cells and other noncancerous components of GC. Surprisingly, these factors could be involved in gastric carcinogenesis. Cytokines, including interleukin-6 and interleukin-11, or growth factors, such as fibroblast growth factor produced from CAFs, can directly activate GC cells and consequently lead to the development of an aggressive phenotype. Galectin-1 or hepatocyte growth factor can be involved in CAF-derived neovascularization in GC. In addition, recent studies showed that CAFs can affect tumor immunity through M2 polarization of tumor-associated macrophages. Finally, the current study aimed to introduce several inhibitory agents and evaluate their suppressive effects on CAFs in patients with GC progression. However, further studies are required to evaluate their safety and select appropriate patients for application in clinical settings.

## 1. Introduction

Gastric cancer (GC) is one of the most common malignancies worldwide and is a major cause of cancer-related mortality [1]. The standard treatment for GC without distant metastasis is resection of the stomach and proper lymph node dissection, and postoperative systemic chemotherapies are recommended for stage II or III [2, 3]. Despite standard treatment for patients with resectable GC, patients with advanced stage GC still show poor prognosis; therefore, the 5-year overall survival rate of patients with stage III was about 20%–40% [4–6]. Meanwhile, the therapeutic option for GC patients with distant metastasis (stage IV) or patients with recurrence after resection is systemic chemotherapy with multidrug regimens, but the outcomes are poor with a reported mean survival time of about 10 months or less [7–10]. Recently, a variety of molecularly targeted agents has been proposed to enhance the survival rate. However, most clinical trials either have not shown a survival benefit, except for trastuzumab and ramucirumab as combined agents with palliative chemotherapy, or are still ongoing [11]. The limited benefit of treatments for GC is increasingly attributed to the tumor stroma, including extracellular matrix (ECM), fibroblasts, immune cells, and microvasculature, as it is well known that GC has a profuse, noncancerous proportion that contributes to GC progression [12–14].

Among these various components in the tumor stroma, cancer-associated fibroblasts (CAFs) have been suggested to play a key role in tumor development [15]. CAFs remarkably influence the tumor microenvironment (TME) via the secretion of cytokines, chemokines, and growth factors [16, 17]. Those secreted proteins enhance cellular migration, alter the metabolism of epithelial tumor cells [18, 19], control the metabolic flexibility of cancer cells [20], and play a significant role in the development of resistance to therapeutic agents [21, 22]. The function of CAFs in cancer progression has been explored in various* in vitro* experimental models using CAFs isolated from primary human solid tumor tissues [23]. Usually, the cancer cells were cocultured with CAFs, which have shown increased migration, invasion, and survival ability, and tumorigenesis of cancer cells was compared with those cocultured with normal fibroblasts [23–25]. Not only* in vitro* but also* in vivo* experiments have confirmed that CAFs advance tumor growth and promote metastasis of cancer cells when coinjected into murine xenograft models [23, 26]. In spite of these results, the unique makers of CAFs and main contributors according to the type of cancer have not been clarified. There have been several well-established indicators of CAFs, such as platelet-derived growth factor *α* (PDGRF*α*), PDGFR*β*, and alpha-smooth muscle actin (*α*-SMA) [15]. However, these markers are typically expressed only in a fraction of fibroblasts within the tumor and are not specific to CAFs. In addition, CAF-derived contributors involved in cancer progression could differ depending on the type of cancer, because they have different carcinogenesis and progression mechanism. Therefore, the mechanisms of communication between CAFs and specific types of cancers need to be investigated.

Based on those characteristics of CAFs in solid cancers, previous studies have shown that the histologic accumulation of CAF in various types of cancers (colon, esophagus, breast, and liver cancer) could be a poor prognostic maker [27–30]. In GC, type IV of traditional Borrmann's classification has a profuse fibrotic stroma showing poor prognosis due to high recurrence in the peritoneum [31–33]. Those clinical studies have implied that CAFs accumulated in GC tissues might enhance the progression and metastasis of GC. In addition, the high throughput gene expression profiling in GC tissues revealed that the tumors with a high expression of “stroma signature” genes contained a high proportion of fibrotic stroma including CAFs and could be a surrogate marker for predicting the prognosis of GC patients [34]. Our previous study also investigated if the accumulation of fibroblast in a specific subtype of GC, signet ring cell carcinoma, was related to the clinical outcomes [35]. In this study, a higher proportion of CAFs, which was evaluated by immunohistochemical staining for *α*-smooth muscle actin and Masson's trichrome staining for stromal collagen, was significantly related to poor prognosis than a lower proportion of CAFs. Taken together, it would be easily assumed that CAFs have a big impact on the GC progression, due to their direct effects on cancer cells or indirect effects on other ecosystems within malignant tumors. The former could induce the stemness or metastatic potency of cancer cells through paracrine or direct contact, while the latter could control noncancerous microenvironments such as angiogenesis or tumor immunity.

To explore the function of CAFs in malignant tumors through experimental models, CAF should be steadily isolated from bulky tumors. To date, several methods to isolate fibroblasts from GC tissues have been established and isolated fibroblasts were confirmed by expression of specific markers [35–38]. For example, the fresh tissues harvested from patients were immediately moved into a clean bench and were cut into small pieces in a culture dish. After mincing with scalpels, a coverslip was placed over the tissue forming a sandwich. Fibroblasts usually outgrew in a monolayer and were subsequently collected [35]. However, because those cells were not immortalized, most researchers used the fibroblasts with low passage number for subsequent experiments.

We aimed to provide an update on the mechanism of CAF-induced GC progression in the view of tumorigenesis, invasion and metastasis, angiogenesis, and tumor immunity. We also aimed to introduce the potential therapeutic strategies that can target the effect of CAFs on the GC cells.

## 2. Origin of CAFs Accumulated in GC

Although CAFs are the predominant cell type within the tumor stroma of various solid cancers, the origin of CAFs is not fully understood. Previous studies have suggested some candidates for the origin of CAFs such as fibroblast in normal tissues [63], specific cells around vessels such as pericytes and vascular smooth muscle cells [64], endothelial cells [65], and bone marrow-derived stem cells [66]. In GC, a pericyte was suggested as one of the origins of CAFs [67]. Here, GC cells could secrete exosomes, which could induce the transition of pericytes into CAFs, but this experimental result was not proven in GC patients' samples. Bone marrow-derived stem cells have also been proposed as the origin of CAFs in GC [68]. This result was proven in the tissues harvested from patients with secondary GC who have previously undergone bone marrow transplantation for various hematologic diseases. Another study insisted that CAFs could be induced from normal resident fibroblasts of the stomach by stimulation of TGF-*β* derived from the scirrhous GC cells [69].

However, in other solid tumors such as those of the head and neck, breast, and pancreas, recent studies show that CAFs contained in one tumor were heterogeneous, presenting different gene expression patterns and a variety of functions [21, 70, 71]. These results imply that CAFs in GC may also include various subtypes that originated from multiple sources, and it would be important to determine which subtype has a crucial role in GC progression. To the best of our knowledge, no study has evaluated the heterogeneity of CAFs in GC; hence, it should be investigated in future studies.

## 3. Role of CAF in Gastric Carcinogenesis

Gastric carcinogenesis is a very complicated process. Because high-throughput genetic profiling in GC tissues did not reveal driver mutation during gastric carcinogenesis [72], the role of environmental factors such as infection and food could be emphasized [73]. Most studies have proposed that those factors could enhance precancerous inflammation in the gastric mucosa, which can lead to GC [74, 75].

Although the role of CAF during gastric carcinogenesis has been rarely reported, several candidates derived from fibroblasts have been suggested as the contributors to the occurrence of chemically induced GC in murine models [76, 77]. In one of those models, GC developed in Lewis rats provided with drinking water with N-methyl-N'-nitro-N-nitrosoguanidine [77]. During carcinogenesis in this rat model, SPARC-stained fibroblasts appeared in the interstitial portion of early initiation stage of stomach tumors in the test rat; however, this was not observed in the control rat. These results suggested that SPARC-expressing fibroblasts probably contributed to GC development. Hiroto K et al. studied the role of CAFs on carcinogenesis using N-methyl-N-nitrosourea- (MNU-) induced gastric tumorigenesis mouse model [76]. In this study, compared with normal gastric tissues, IL-6 expression in GC was significantly increased, and IL-6 knockout mouse had a lower incidence of MNU-induced GC than wild-type mice. These results imply that IL-6 induced from CAFs has an important role during gastric carcinogenesis.

## 4. Role of CAF in GC Invasion and Metastasis

The invasion and metastasis of cancer cells have long been the causes of death and great challenges for GC patients even after undergoing complex clinical treatments [78]. The poor prognosis and low survival rate of GC patients are mainly due to metastasis [45], and almost 60% of GC deaths are due to peritoneal recurrence [50]. However, the specific mechanisms of GC metastasis have not been clarified.

The epithelial-mesenchymal transition (EMT) is a biological process by which epithelial cells lose their cell polarity and cell-cell adhesion, gain migratory and invasive capacity, and become resistant to apoptosis. Moreover, the EMT increases the production of components of ECM and gains the invasive properties to become mesenchymal stem cells, which play an important role in the initiation of metastasis during cancer progression [47]. In GC, high-throughput molecular analysis revealed that the expression of EMT gene signature in primary GC was significantly related to poor prognosis [79]. As described earlier, because CAFs were known to advance tumor cell metastasis and invasion by overexpression of a variety of factors that can enhance EMT phenomenon [39], the exploration of communication mechanism between GC cells and CAFs could be crucial in the field of GC metastasis research.

One study described that the effect of CAFs on increased migration of GC cells was more significant than normal tissue-associated fibroblasts. This study suggested that microRNA-106b is a CAF-specific maker and has a crucial role in the reinforcement of phosphatase and tensin (PTEN) signaling in GC cells [40]. While this study did not indicate the exact communicators between GC cells and CAFs, Wu X et al. [41] showed that GC-derived CAFs secrete significant quantities of IL-6, which can induce EMT phenomenon and increase migration of GC cells through activation of Janus kinase 2/signal transducers and activators of transcription (JAK2/STAT3) pathway in GC cells. In addition, they showed that deprivation of IL-6 by inhibiting the JAK/STAT3 pathway with a specific inhibitor markedly diminishes these phenotypes in GC cells induced by CAFs. Another secreted factor such as fibroblast growth factor 9 (FGF-9) could be suggested as a communicator between GC cells and CAFs [42]. This study showed that the CAFs isolated from GC tissues could secrete FGF-9 into the extracellular area under the regulation of miR-214 and the secreted FGF-9 could induce EMT in GC cells. CAF-derived exosomes could be a key player in the communication between GC cells cultured from scirrhous type GC, which is a subtype of GC with abundant fibrotic stroma [37] because exosomes are cell-derived vesicles containing functional biomolecules that can be transferred to recipient cells [43]. In particular, CD9 is a specific marker of exosomes that originated from CAFs, and CD9 exosomes from CAFs could increase the migration and invasion ability of GC cells.

Taken together, the evidence suggests that CAFs may play a pivotal role in the migration and invasion of GC cells. In addition, other factors such as stromal derived factor 1 (SDR1), CXCL12, and interleukin 11 have been suggested as CAF-derived inducers for migration and invasion of GC [36, 38, 44, 46, 48, 49, 80, 81], and their mechanisms are listed in Table 1. The mechanisms associated with CAF-induced motility of GC cells could be a novel target in the treatment of GC.

## 5. Role of CAF in Angiogenesis of GC

Pathological angiogenesis is a hallmark of cancer [82]. Growth, invasion, and metastasis of malignant tumors depend on neovascularization that is controlled by proangiogenic and antiangiogenic elements [83, 84]. Past studies have shown the positive correlation between the expression of factors related to tumor angiogenesis and poor clinical outcomes of GC patients [85, 86]. Moreover, antiangiogenic agent, a monoclonal antibody VEGFR2 antagonist, is one of the few targeted agents showing clinical benefit in metastatic GC patients [87]. A plethora of factors have been proposed as contributors to angiogenesis, but major factors should be clarified before administering novel targeted agents to block GC angiogenesis.

Increasing evidence has shown that chemokine secretion by CAFs may support the recruitment of bone marrow-derived angiogenic cells [58]. CAFs may be a major source of angiogenic factors [88]. In GC, galectin-1 [89] and hepatocyte growth factor (HGF) [90] have been proposed as CAF-derived secretory proteins, which contribute to GC angiogenesis. Galectin expression in CAFs was positively related to increased expression of endothelial cell marker, CD31 [89]. Ding X et al. [90] discovered that the phosphorylation of Akt and ERK1/2 was increased in GC cells treated with HGF and cocultured with CAFs. Both Akt inhibitors and ERK1/2 inhibitors reduced the angiogenic and vasculogenic abilities of HGF. However, these results have been confirmed using an* in vitro* angiogenesis assay (tube formation assay). To elucidate the correlation between CAFs and GC angiogenesis, the inhibitory effects of CAF-derived proteins on GC angiogenesis should be investigated in GC animal models for clinical application.

## 6. Role of CAF in GC Resistance to Chemotherapy

Chemotherapies for GC have shown some clinical effects; however, some patients still show progression and recurrence after chemotherapy in clinical settings, and there are many obstacles to overcome this issue. One of the commonly reported reasons for failed chemotherapy in clinics is the occurrence of drug resistance. Unfortunately, the underlying mechanism of multidrug resistance in GC remains unclear.

Previous studies evaluating chemotherapy resistance have focused on the tumor microenvironment. In particular, cancer cell–ECM interactions, CAF–ECM adhesion, and cytokine or chemokine-mediated signaling pathways have been considered as TME-related resistance to chemotherapy [91, 92]; CAFs may have a major role in those mechanisms. CAFs have been confirmed to regulate chemoresistance by secreting cytokines, including stromal cell-derived factor-1*α*, IL-6, and IL-7 [93–95], and may also increase intratumoral interstitial fluid pressure, thus indirectly inhibiting the uptake of anticancer drugs [96]. However, the role of CAF in chemoresistance has not been clarified.

Only one study was published describing that CAFs secreting IL-11 could contribute to resistance to combined chemotherapy regimens in GC cells by activating gp130/JAK/STAT3/Bcl signaling pathway [97]. Recently, our group investigated the GC CAF-specific secretory protein involved in chemoresistance [98]. Through the analysis of transcriptome between fibroblasts from paired normal gastric and GC tissues, IL-6 was suggested as a CAF-specific cytokine. In addition, transcriptome data and immunohistochemical staining for GC tissues revealed that IL-6 was usually expressed in the fibrotic stromal cell. CAF-derived IL-6 could induce resistance to 5-FU or cisplatin in various experimental models, such as* in vitro* and* in vivo* xenograft, and tocilizumab, a monoclonal antibody that inhibits the binding of IL-6 to its receptor, effectively suppressed the development of drug resistance. If those results were applied in the clinical setting, it could have prevented the occurrence of chemoresistance in GC patients.

## 7. Role of CAF in Tumor Immunity of GC

The Cancer Genome Atlas (TCGA) project for GC revealed four molecular subtypes [72]; among them, Epstein–Barr virus- (EBV-) positive and microsatellite instability subtype was associated with high-density tumor-infiltrating lymphocytes and showed a better prognosis compared with other subtypes [99]. Some previous studies have reported that infiltrating immune cells had an effective host immune response against GC cells [100, 101]. Taken together, the tumor escape from immune response could deteriorate the outcome of GC patients; therefore, this mechanism could be a good target to improve the patients' prognosis. However, the exact mechanisms involved remain unknown.

CAFs produce a plethora of cytokines and chemokines potentially contributing to tumor immunity at various stages of cancer progression. The direct or indirect effects of IL-6, IL-8, IL-10, TGF-*β*, C-C motif chemokine ligand 2 (CCL2), C-X-C motif chemokine ligand 9 (CXCL9), and CXCL10, but not limited to those, on tumor immunity in patients with oral, breast, and pancreatic cancer have been investigated [102–104]. The role of CAFs in the regulation of tumor immunity is seldom reported in GC. However, recent studies show that CAFs were deeply involved in M2 polarization of macrophage suppressing immune clearance [105, 106]. CAFs could induce M2 polarization in tumor-associated macrophage (TAM); it has been well reported that the accumulation of M2 macrophage was significantly related to the poor survival of GC patients [107] and M2 macrophages directly induced invasion and metastasis of GC cells or indirectly reduced immune response within GC tumors. The proportion of CAF in the deep portion of the primary GC is higher than that in the superficial layer, which positively correlates with the increased number of M2 macrophages [105]. Other studies reported that neurooncological ventral antigen 1 (NOVA1), a marker of activated CAFs, was suppressed in GC microenvironment including CAFs, and NOVA1 suppression was significantly correlated with immune dysfunction such as an accumulation of M2 macrophage [106]. Although several secretory proteins such as macrophage colony-stimulating factor (M-CSF) [108], interleukin 33 (IL-33) [109], CCL2, and interleukin 6 (IL-6) [110] have been suggested as stimulators derived from CAFs for M2 macrophage in esophageal and pancreatic cancers, it has never been reported in GC and should be discovered to be applied for clinical setting in the future.

## 8. CAF-Targeting Agents

As the role of CAFs in the progression of solid tumors becomes clearer, several therapeutic approaches to inhibit the function of CAFs have been suggested as novel agents.

Some of those CAF-targeting agents have been already applied in clinical settings in patients with various malignant or nonmalignant diseases, but they were not used as CAF inhibitors. Nilotinib is an inhibitor of the c-KIT receptor and is effective in the treatment of chronic myeloid leukemia, melanoma, and gastrointestinal stromal tumors [111–113]. Aside from c-KIT receptors, nilotinib also inhibits other receptor tyrosine kinases such as platelet-derived growth factor receptors (PDGF-R*α* and PDGF-R*β*) or discoidin domain receptors (DDR1 or DDR2) [52, 114]. A previous study reported that PDGF-R was expressed in CAFs, not in cancer cells [60]. As activated PDGF-R signaling pathway in tumor stroma can increase the proliferation of cancer cells [57] and stimulate GC angiogenesis [61], nilotinib could be used as a potential inhibitor for GCs with a profuse fibrotic stroma [51]. Tocilizumab has been clinically used in several patients with rheumatic disease as an inhibitor of a pleiotropic cytokine such as IL-6. This drug has been proposed as a potential inhibitor of GC cells and CAFs. The* in silico* analysis using TCGA database of GCs revealed that low expression of both* IL-6* and* IL-6R* genes was significantly related to improved survival of GC patients and the* in vivo* experiments described that tocilizumab could efficiently reduce tumor growth in xenograft models of GC cells mixed with CAFs [53].

Several natural products have been proposed as suppressors of CAF activity. Astragaloside IV, the main component of nontoxic Chinese herb, inhibited cellular migration by reducing the ability of CAFs to promote GC cell migration and invasion through regulation of microRNA such as miR-214 in the nontoxic low dose [54]. Paeoniflorin, the principal bioactive component of Radix Paeoniae Rubra, inhibited the secretion of IL-6 from CAFs and consequently inhibited the migration- and invasion-promoting capacities of GC CAFs [55].

Other agents have been evaluated to determine their efficacy in suppressing the migration- and invasion-promoting capacities of GC CAFs through various preclinical models [46, 56, 59, 62, 115–118]; these agents are listed in Table 2. However, future studies are required to determine the toxic effects and indications of those agents.

## 9. Conclusion and Future Perspectives

**Figure 1:**
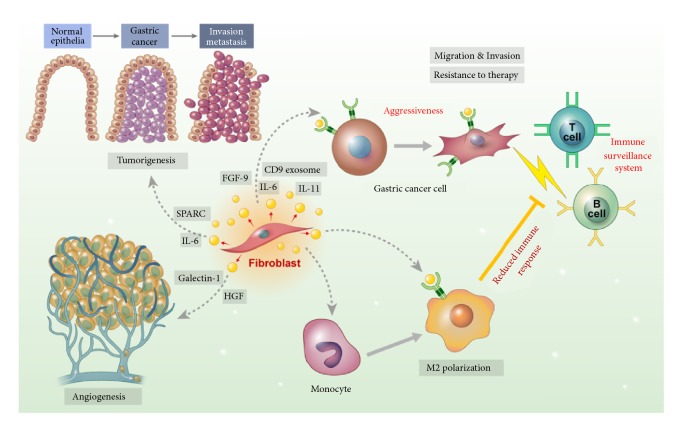
Schematic image for role of cancer-associated fibroblasts in tumor microenvironment of gastric cancer.

Studies have shown that the CAFs are an important component in the TME of GC, and previous studies revealed the potential effects of CAFs including carcinogenesis, metastasis, invasion, angiogenesis, resistance to therapy, and tumor immunity in various GC models (Figure 1). However, the inhibitory mechanism of CAFs on GC cells as well as TME has not been applied in GC treatment. Moreover, the specific markers and origin of CAFs remain controversial. Recent advanced technologies for single-cell transcriptome profiling have uncovered spatial, functional, and genomic heterogeneity of cancer cells and associated host cells in TME [119]. The single-cell RNA-sequencing for lung [120], pancreas [121], and colorectal cancer [122] revealed that CAFs in solid tumors have molecular and functional intra- and interheterogeneity and suggested specific CAF subpopulations as targets for cancer treatment. However, to the best of our knowledge, there has been no report that studies CAFs heterogeneity through the single-cell molecular profiling in GCs. Considering the functional role of CAFs in GCs, further studies evaluating CAF heterogeneity are warranted to determine the critical CAF subtype that expresses specific targets for GC treatment.

## Figures and Tables

**Table 1:** Mechanisms involved in CAF-induced migration or invasion in gastric cancer.

Author	Year	CAFs derived contributor	Regulation of contributor in CAFs	Receptor in cancer cell	Activated signaling pathway in cancer cell	Experimental models	Reference
Yu B et al.	2013	Transgelin (TAGLN)			Metrix metalloproteinase-2 (MMP-2)	Transwell migration	[39]
						Invasion assay	
						Xenograft model	

He XJ et al.	2014	Galectin-1 (Gal-1)		Integrin receptor	Gal-1/Integrin *β*1 axis	Transwell migration	[40]
						Invasion assay	

Kasashima H et al.	2014	Lysyl oxidase-like 2 (LOXL2)		Integrin receptor	Src/focal adhesion kinase (FAK) pathway	Wound healing assay	[41]
						Invasion assay	

Izumi D et al.	2016	CXCL12		CXCR4	Integrin *β*1/FAK pathway	Invasion assay	[42]
						Real-time imaging	

Qiao J et al.	2016	Stromal cell derived factor 1 (SDF1; CXCL12)	Serum response factor (SRF)	CXCR4	SDF1-CXCR4 axis	Transwell migration	[43]
						Wound healing assay	
						Invasion assay	
						Xenograft model	

Wang X et al.	2017	Lumican			Integrin *β*1/FAK pathway	Transwell migration	[44]
						Invasion assay	
						xenograft model	

Wu X et al.	2017	Interleukin-6 (IL-6)		Cell-surface type I cytokine receptor complex	JAK2/STAT3 pathway	Transwell migration	[45]
						Peritoneal xenograft	

Ding X et al.	2018	HGF/IL-6		c-MET/IL-6R	JAK2/STAT3/twist1 pathway	Transwell migration	[46]
						Invasion assay	
						Xenograft model	

Miki Y et al.	2018	CD9-positive exosomes			MMP-2	Wound healing assay	[47]
						Transwell migration	

Suzuki M et al.	2018	TGF‐*β*1				Invasion assay	[48]
						Wound healing assay	

Wang X et al.	2018	IL-11			JAK/STAT3 and MAPK/ERK pathway	Transwell migration	[49]
						Invasion assay	

Wang R et al.	2019	Fibroblast growth factor 9 (FGF9)	Downregulation of miR-214			Transwell migration	[50]

**Table 2:** Agents for inhibition of communication between CAFs and GC cells.

Author	Year	Agents	Origin	Cell lines	Target molecules	Tools	Outcomes	Reference
Yashiro M et al.	2003	Tranilast	Chemical	OCUM-2D	Matrix metalloproteinse-2 Transforming growth factor-beta 1	Invasion assay	Invasiveness	[51]

Onoyama M et al.	2013	Nilotinib with Everolimus	Chemical	TMK-1 MKN-1 KKLS	PDGF-R tyrosine kinase and Mammalian target of rapamycin	Xenograft	Tumor growth Angiogenesis Metastasis Stromal reaction	[52]

Hara M et al.	2016	Itraconazole	Chemical	HT-29 (colon) MKN-28 MKN-45	Mitogen-activated protein kinase Protein S6	Flow cytometry Angiogenesis assay Xenograft	Angiogenesis Tumor growth	[53]

Izumi D et al.	2016	AF-310-NA AMD3100 PF-573,228	Antibody Chemical Chemical	AGS KATOIII	CXCL12 CXCR4 Focal adhesion kinase	Real-time imaging Invasion assay	Migration Invasiveness	[42]

Jin H et al.	2017	7rh	Chemical	MKN-45 MKN-74	Discoidin domain receptor 1	Spheroid culture Xenograft	Tumorigenesis	[54]

Pang T et al.	2017	Fiber-modified hexon-chimeric oncolytic adenovirus	Adenovirus	MKN-45	Fibroblast activation protein (for CAF-specific infection)	Cell viability assay Xenograft	Tumor growth	[55]

Wang Z et al.	2017	Triptonide	Natural products (*Tripterygium wilfordii*)	BGC-823	microRNA-149 ↑ microRNA-301a ↓	Colony formation assay Wound healing assay Invasion assay	Tumorigenesis Migration Invasiveness	[56]

Wang ZF et al.	2017	Astragaloside IV	Natural products (A*stragali Radix*)	BGC-823	microRNA-214 ↑ microRNA-301a ↓	Proliferation assay Wound healing assay Invasion assay	Proliferation Migration Invasiveness	[57]

Ding X et al.	2018	LY294002 U0126	Chemical		AKT ERK 1/2	Angiogenesis assay	Angiogenesis	[58]

Dong R et al.	2018	Polyphyllin I	Chemical	MKN-45	Fibroblast activation protein alpha Hepatocyte growth factor	Proliferation assay Xenograft	Proliferation Tumor growth	[59]

Karakasheva TA et al.	2018	Tocilizumab	Antibody	ESCC EAC	IL-6 receptor alpha	3D culture 3D organotypic culture Xenograft	Tumorigenesis	[60]

Wang ZF et al.	2018	Paeoniflorin	Natural products (*Radix Paeoniae Rubra*)	AGS	microRNA-149 Interleukin 6	Wound healing assay Invasion assay	Migration Invasiveness	[61]

Chen G et al.	2019	Metformin	Chemical		Calmodulin-like protein 3	Proliferation assay	Tumor promotion	[62]
